# Predicting and Interpreting Spatial Accidents through MDLSTM

**DOI:** 10.3390/ijerph18041430

**Published:** 2021-02-03

**Authors:** Tianzheng Xiao, Huapu Lu, Jianyu Wang, Katrina Wang

**Affiliations:** 1 Institute of Transportation Engineering and Geomatics, Tsinghua University, Beijing 100084, China; xtz16@mails.tsinghua.edu.cn (T.X.); luhp@tsinghua.edu.cn (H.L.); 2 Division of Biosciences, University College London, London WC1E 6BT, UK; katrina.wang.19@ucl.ac.uk

**Keywords:** traffic accident, MDLSTM, spatial, interpretation

## Abstract

Predicting and interpreting the spatial location and causes of traffic accidents is one of the current hot topics in traffic safety. This research purposed a multi-dimensional long-short term memory neural network model (MDLSTM) to fit the non-linear relationships between traffic accident characteristics and land use properties, which are further interpreted to form local and general rules. More variables are taken into account as the input land use properties and the output traffic accident characteristics. Five types of traffic accident characteristics are simultaneously predicted with higher accuracy, and three levels of interpretation, including the hidden factor-traffic potential, the potential-determine factors, which varies between grid cells, and the general rules across the whole study area are analyzed. Based on the model, some interesting insights were revealed including the division line in the potential traffic accidents in Shenyang (China). It is also purposed that the relationship between land use and accidents differ from previous researches in the neighboring and regional aspects. Neighboring grids have strong spatial connections so that the relationship of accidents in a continuous area is relatively similar. In a larger region, the spatial location is found to have a great influence on the traffic accident and has a strong directionality.

## 1. Introduction

According to the report published by the World Health Organization (WHO), road traffic crashes result in the deaths of approximately 1.35 million people around the world each year and leave between 20 and 50 million people with non-fatal injuries [1]. Factors affecting traffic accidents can be divided into subjective and objective aspects at the macroscopic level. The objective aspects mainly include regional characteristics, road network characteristics, climate characteristics and so on. The subjective aspects mainly include human operation errors, violations of regulations, negligence, vehicle technical reasons and so on. The involvement of multiple influencing factors complicates the prediction and analysis of traffic accidents, and makes it difficult to strip out the influence of any one of these factors. Although current research is centred on quantitatively analyzing the conditions of different influencing factors and elucidating the most influential factors [2], gaps in this area of knowledge remain. 

The revelation of significant spatial auto-correction in traffic accidents from spatial analysis brought an inspiration: since the multiple causes of traffic accidents are also spatial aggregates, the spatial influence on such traffic accidents must contain many valuable factors that are not directly observed, hence, local land use characteristics and spatial correlation are analyzed concurrently in this paper, using the multi-dimensional long-short term memory neural network model (MDLSTM). The method greatly improves the accuracy of traffic accident prediction by responding to multi-variate inputs with non-linear relationship. More indicators that make comparisons existing research in the model input and output are taken into account, which is also the advantage of the MDLSTM model. In addition, this method can capture the relationship between some variables that traditional models consider to be unrelated.

In comparison with the spatial regression models, this study also considers potential accidents which traffic accidents are based on. The potential accidents not only show the occurrence or numbers of traffic accidents, but also indicate the date, time, isolation form and the cross-sectional location of traffic accidents. Due to the lack of explicit prior information, this complex regulation is modelled using the neural network model.

## 2. Literature Review

Researchers in the field of traffic safety have been found to trace the source of traffic demand to excavating the causes of traffic accidents. Decades ago, among objective factors, researchers also focused on the impact of road network layout, road and traffic design, traffic control, active risk management and environmental conditions on traffic safety, and the problem of traffic accidents caused by land use. There were few studies on the issue of traffic accidents related to land use, and the topic is becoming more and more critical currently.

To analyze the impact of objective factors on the location of traffic accidents, it must been found that the spatial characteristics and differences of traffic accidents. For cities, the fundamental spatial differences include three parts: First are the land use characteristics. Land use characteristics lead to the differences in traffic demand and form the spatial distribution of Origin-Destination (OD) pairs. For example, tidal traffic conditions are more likely to be occurred around educational and office plots since the demand is concentrated in the morning and evening peaks. However, the traffic conditions around medical plots are more likely to be affected by emergencies. Second is the location feature. For a general single-center city, the traffic demand in the central area is often greater than that in the suburban areas, which is determined by the spatial agglomeration of the choosing behavior of individuals. Third is the transportation supply represented by road facilities. The quality of road facilities, road network density, design and construction level, etc. are often indirectly related to spatial location, but they have the characteristics of continuous stability. As traffic demand continues to increase, traffic accidents are also gradually increasing. In some special region, traffic accidents are observed to have strong spatial autocorrelation, which may lead to an inspiration that there are some gathered characteristics influencing the accident in the similar way as the statement above.

### 2.1. Spatial Analysis of Traffic Accidents

Spatial analysis of traffic accidents have been used in designing road safety measures for decades, in order to determine how crashes are affected by the neighboring locations, how the influence of parameters varies spatially, and which locations require more urgent interventions [2]. Previous researches have focused on the use of different spatial unit [3], different modelling approaches [4,5,6], and the corresponding study design characteristics, including traffic, road environment and area parameters and spatial aggregation approaches, where geographically weight regression [7,8], Bayesian models [9] and machine learning methods [10,11] were applied.

In 1995, Levine et al. [12] studied the distribution of traffic accidents on the main road network in Honolulu, Hawaii, and found that traffic accidents are spatial correlated. Levine et al. [13] used a modified multiple linear regression method to establish the statistical relationship between the total number of traffic accidents in the block (Census-Block-Group) and the corresponding population, employment, and road traffic flow. Spatial statistical methods have been applied more frequently in macro safety analysis. Erdogan [4] used the Kernel Density Estimation (KDE) method to calculate the density of hotspots along the Kaduna-Abuja route. The results determined 222 accidents on the road between 2010 and 2014 and 8 different hotspots along the way, including Gonin Gora, Toll stations, Sabon Gaya, etc. Quddus [14] analyzed the traffic accidents at the Ward-level using the Bayesian spatial statistical model built for London. Lord et al. [15] proposed the application of negative binomial regression to establish a traffic safety model with characteristics of the planned road network, which was used to analyze the safety of planning schemes in traffic planning. Lovegrove et al. [16] analyzed the feasibility of applying the macro-safety model to evaluate traffic improvement schemes in the traffic analysis zone (Traffic-Analysis-Zone, TAZ) in a case study.

### 2.2. Influencing Factors of Traffic Accidents

Different factors have different effects on traffic accidents. Previous studies on influencing factors of traffic accidents mainly focused on the attributes of personnel [17], vehicles [18], roads [19] and environment [20]. For example, Liu and Fan took traffic accidents from 2005 to 2013 in North Carolina as a sample and found drunk driving behaviors had huge impact on traffic accidents [21]. Kelley et al. studied the crash data in CIREN database from 1998 to 2012 and found side impact could be an important influencing factor on traffic accidents [22]. Cheng et al. researched on traffic accident data from San Francisco from 2008 to 2013 and found severe weather could be related to serious traffic accidents [23]. None of the existing studies looked at the causes of traffic accidents from the aspects of urban zoning differences [24], road network topology [25], etc. In our study, factors, such as plot ratio, point of interest and congestion ration representing urban zoning differences and road network topology are used to find more specific causes of traffic accidents.

Researchers in the field of traffic safety have been found to use spatial distribution as clues to track the causes of traffic accidents and focused on environmental factors. Decades ago, among objective factors, researchers also focused on the impact of road network layout, road and traffic design, traffic control, active risk management and environmental conditions on traffic safety, and the problem of traffic accidents caused by land use. There are little studies on the issue of traffic accidents related to land use, and this topic is becoming increasingly important.

## 3. Materials and Methods 

### 3.1. Data

#### 3.1.1. Data Sources

The land use properties and traffic accident data both come from the City of Shenyang in China, which is also the area of study in this paper. The land use dataset is compiled from the point of interest (POI) data, the evening peak traffic flow data and road maps, which are collected from Open Street Map (OSM). Since POI data focus more on commercial service facilities, such as catering and entertainment, the residential data of POI are verified with the residential area information on the Anjuke platform. Fourteen basic types of POI are: Catering, Hotel, Shopping, Life Services, Tourism, Leisure and Entertainment, Sports and Fitness, Education, Medical, Transportation Facilities, Finance, Residential, Companies, and Government Organizations. The POI and evening peak traffic flow data are gathered from the Baidu Map API (Baidu, Beijing, China), and it is the average of the traffic state data of the evening rush hours from 14 June 2019 to 21 June 2019. Table 1 provides an overview of the land use dataset.

The accident dataset is based on the statistics of traffic accidents in Shenyang from Jan 2015 to Dec 2017, an overview is provided in Table 2. The following fields are included: text description of the accident location, date and time, isolation of the road and cross-sectional location in the road. These indicators are all turned to a digital form to accurately model the occurrence or characteristics of traffic accidents.

#### 3.1.2. Distribution of Accidents Characteristics

**Figure 1:**
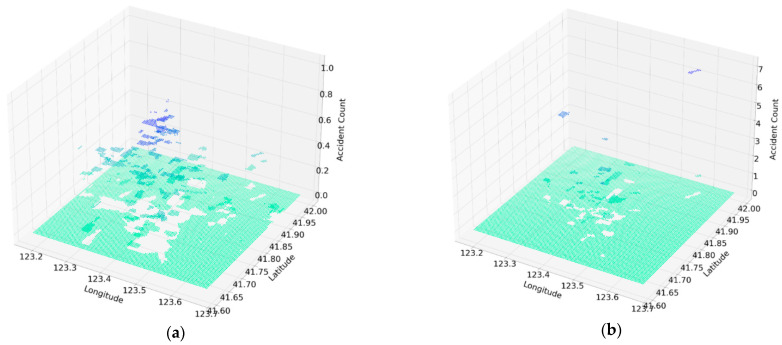
(**a**) The spatial distribution of the “traffic accident counts” indicator; (**b**) The spatial distribution of real traffic accidents.

The text description of accident location is matched to their latitudinal and longitudinal positions through the Baidu Map API, so that all accidents can be traced. In order to describe the traffic accidents from a macroscopic perspective, traffic accidents within 3000 m of each grid cell will be recorded as “traffic accident counts” indicator of the grid cell. The height values in the right-hand side diagram of Figure 1a represents the location of the accident. The values show the number of traffic accidents that took place within a radius of 3000 m from the center of the grid cell, as shown in Figure 1b.

**Figure 2:**
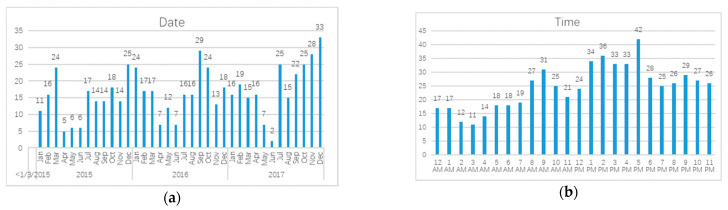
(**a**) The distribution of accident date. (**b**) The distribution of accident time. AM and PM in (**b**) are “ante meridiem” and “post meridiem” which means time period before noon (0:00–12:00) and after noon (12:00–24:00).

The accident date and accident time are processed to linearize the relationship with the accident frequency. The date of the traffic accident is converted to the number of days till winter (represented by the winter solstice on 22nd December). Since Shenyang has more road icing in winter, winter is the season where most traffic accidents occur, as shown by Figure 2a, where the three peaks in the data distribution of traffic accident data corresponds to the three winters in 2015, 2016 and 2017.

In Figure 2b, the accident time data is illustrated in a similar fashion as the accident date data. 13:00–17:00 is the time period when traffic accidents occur frequently, so the time distance to 15:00 is taken as the value of the indicator.

**Figure 3:**
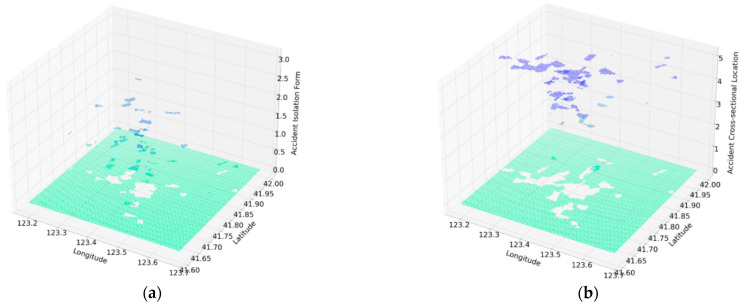
(**a**) The spatial distribution of the isolation form. (**b**) The spatial distribution of the cross-sectional location.

As shown in Figure 3a, the isolation of the road is one of the effective factors influencing traffic accidents. There are 4 levels of isolation of the road: the “Center isolation and motor vehicle-non-motor vehicle isolation”, “Center isolation”, “Motor vehicle and non-motor vehicle isolation” and “None”, each denoted 4, 3, 2 and 1, respectively. Figure 3b shows the cross-sectional location is another key feature in traffic accidents. There are 5 levels of cross-sectional location: “Motor vehicle lane”, “Motor vehicle and non-motor vehicle mixed lane”, “Non-motor vehicle lane”, “sidewalk” and “cross walk”, corresponding to 5, 4, 3, 2, and 1, respectively. The spatial distributions of these two indicators are as follows.

#### 3.1.3. Rasterization

The data processing in this study is trying to connect the traffic accident data with the land use properties. The spatial auto-correlation is included to model the unobvious effect. To achieve this, rasterization is used to break up the land use data into raster shapes. The traffic accident data and raster data are then matched spatially, so that the MDLSTM model can capture the spatial relation between accident and land use.

**Figure 4:**
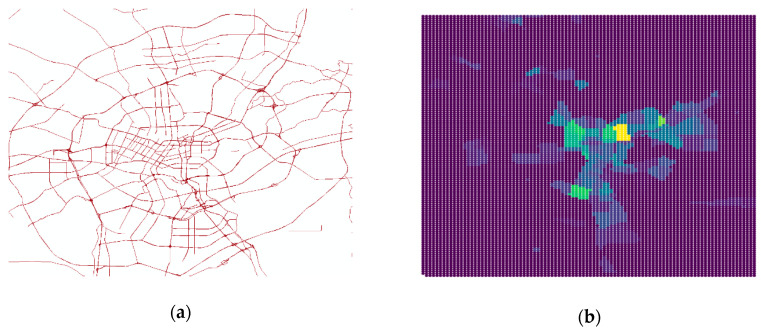
(**a**) Schematic of the urban area in the City of Shenyang with roads. (**b**) The plot ratio data after rasterization.

The location of study of this paper is the urban area in the City of Shenyang, as shown in Figure 4a. Similar to Liu [26] and Yue [27], the rectangular region are rasterized to grid cells at the scale of around 400 m × 444 m, with the usual method [28,29] as shown in Figure 4b. In total, 12,110 grid cells (about 96 rows and 125 columns) were collected in the Shenyang urban area. To speed up learning and convergence when training the model, layer normalization was performed to scale the data into the range [0, 1] as studied by Ba J L [30].

**Figure 5:**
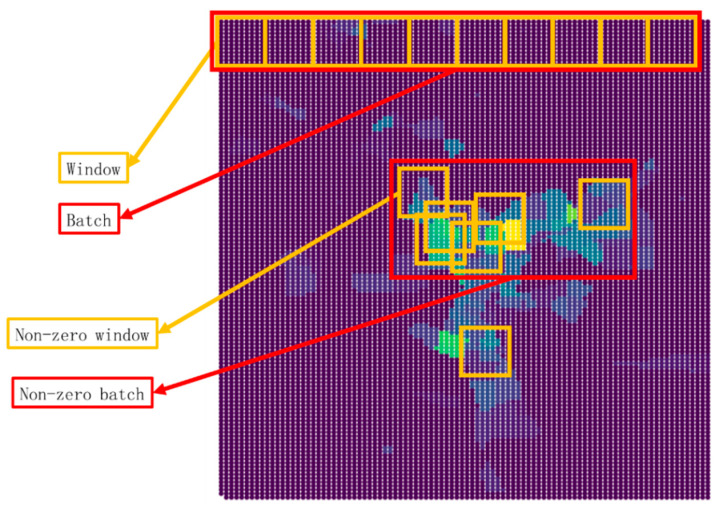
The window sampling and batch splitting.

Data processing has many steps, including map acquisition, data matching, grid transformation, window sampling and batch splitting. After batch splitting, a sliding window (9 × 9 grids) was used to sample the data and reshape them into bi-dimensional tensors, resulting in 10,092 windows in the study area. As shown in Figure 5, the windows were selected by a zero rate index, which means a window is marked as unusable when 80% of the data in the window is missing for lack of information. Among them, 100 randomly selected windows are used as the test dataset, and the remaining 9992 windows are used as the training dataset.

### 3.2. Validation of the Spatial Autocorrelation

The premise of this study is that traffic accidents have significant spatial auto-correlation, which give rise to the assumption that the multiple causes of traffic accidents are also spatial aggregates, and the spatial influence of such traffic accidents contains many valuable factors that are not directly observed. In this section, the spatial auto-correlation is first validated to show that these indicators do have spatial correlation.

The spatial dependency was tested using Global Moran’s I and Global Geary’s C statistics. The results are shown in Table 3. A statistically significant spatial cluster was found, and both results are significant at *p* < 0.001 significance level.

### 3.3. MDLSTM Model

**Figure 6:**
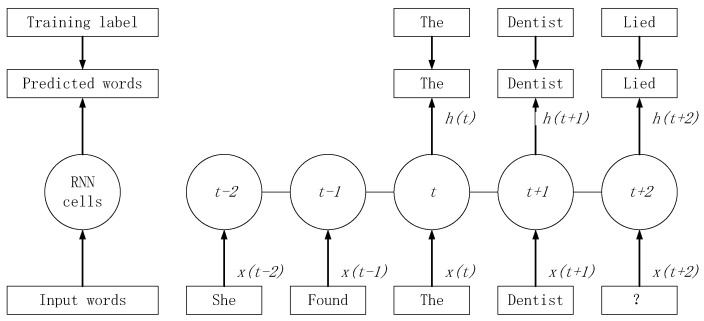
Application of recurrent neural network in natural language processing. “*t*” means the word step while “*t − 1*” means the previous step of “*t*”. The “*h(t)*” means the output of step “*t*” and the “*x(t)*” means the input of step “*t*”. The “?” means the word that needs to be predicted corresponding to the predicting result word “Lied”.

The basic model of MDLSTM is the recurrent neural network (RNN) model developed to simulate the regulation of sequence data. RNN can be widely applied in natural language processing (NLP), since it has the strength of fitting the non-linear relationship between words’ occurrence in a specific location and other words in the context. The advantage of this model is that it can retain the information transferred between distanced words. Take Figure 6 as an example.

**Figure 7:**
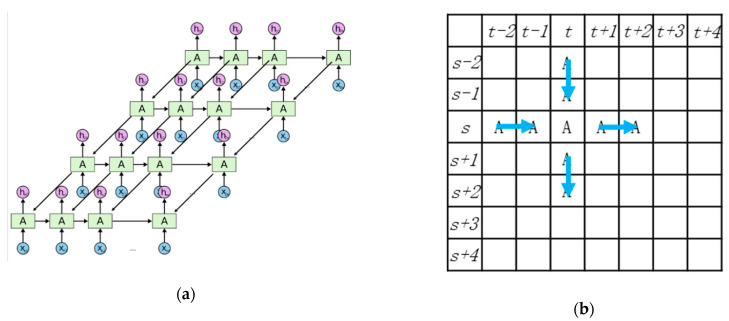
(**a**) The model structure of MDLSTM. (**b**) The application in the accident analysis. ”*t*” and “*s*” shows the coordinate location of the grid cell. For example, the (*t* − 1, *s*) is the left grid cell of (*t, s*). ”A” in this figure shows the MDLSTM cell in a special location, for example the “A” in the *t*th column and *s*th row corresponding to the MDLSTM cell in the same location. The blue arrow means the information transfers from the neighboring grid cell.

The word “Dentist” is the input of the second step (*t* − 1) of the model, and the next word “Lied” is the expected output. In this process, the occurrence of “Lied” is affected by not only the word “Dentist”, but also the previous inputs, such as “The”. MDLSTM is the bi-dimensional version of the developed form of RNN, which has the structure below:

As shown in Figure 7a, the improvements made on the model to a basic RNN are in two aspects. The first is the increases of the long-distance impact through the widely known “Gate” structure, which gave rise to the development of the Long-Short Term Memory neural network model in 1997 [30]. The second is the expansion of the dimension of LSTM in 2007 [31], which made the model more suitable for spatial analysis. In the traffic accident context, every cell in the bi-dimensional network represents a grid cell in the urban area, as shown in Figure 7a.

**Figure 8:**
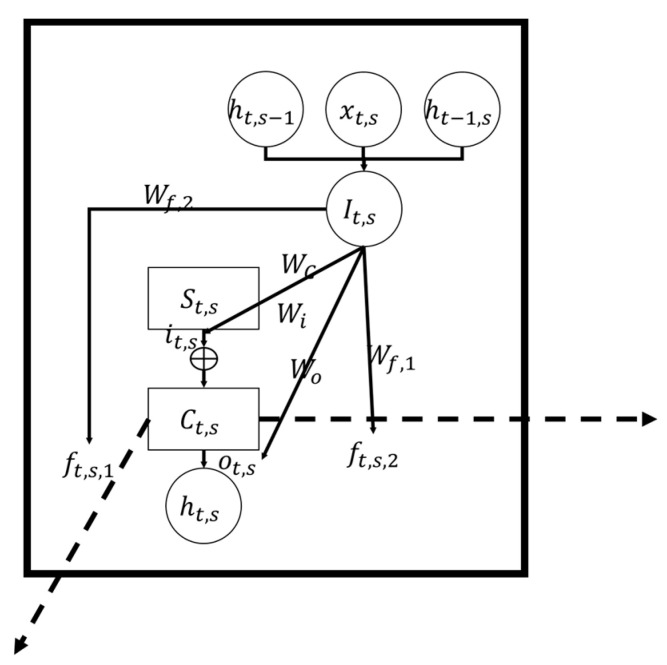
The structure of MDLSTM cell. Arrows in the figure shows the linear transformation from the variable behind the arrow to the variable in front of the arrow.

Influences among the grids cells are further expanded, as shown in the following figures. Figure 8 represents the cell (*t*, *s*) in the MDLSTM model shown in Figure 7a, with the input $x_{t,s}$ and the output $h_{t,s}$. It also represents the grid cell located at (*t*, *s*) in the urban area shown in Figure 7b; since all grid cells have the same trained parameters, the A used are duplicated in every cell. The structure consists of input, output and transfer.

In the urban safety context, the input $x_{t,s}$ is the land use properties, including the plot ratio, number of types of POIs, centrality, distance to the CBD, number of surrounding road sections and the congestion ratio of grid cell located at (*t*, *s*). The output $h_{t,s}$ is the accident characteristics, including the accident counts, date, time, isolation, cross-sectional location of the grid cell (*t*, *s*). Others are intermediate variables, including $S_{t,s}$, $i_{t,s}$, $f_{t,s,j}$, and $o_{t,s}$, which vary between grid cells. Weights and bias, including $W_{C}$, $W_{i}$, $W_{f,j}$, $W_{o}$, $b_{C}$, $b_{i}$, $b_{f,j}$, and $b_{o}$ are the same for every cell in the entire network.

Based on the three steps, which outlines the basic flow of the model, the relationship among the land use properties of every grid cell, the accident characteristics of the surrounding cells, and the accident characteristics of the current cell (*t*, *s*) are as follows:

Input: *S_t,s_* = *tanh*(*W_C_*·*I_t,s_* + *b_C_*) (1)

Transfer: *C_t,s_* = *i_t,s_*·*S_t,s_* + *f_t,s,1_*·*C_t−1,s_* + *f_t,s,2_*·*C_t,s−1_* (2)

Output: *h_t,s_* = *o_t,s_*·*tanh*(*C_t,s_*) (3)

*I_t,s_* = (*x_t,s_*, *h_t−1,s_*, *h_t,s−1_*) (4)

where *S_t,s_* is the state from the local land use. *C_t,s_* is the total state. *tanh* is a commonly used “hyperbolic tangent function” function in the machine learning method. *W_C_* and *b_C_* are the weight matrix and bias matrix of the state *S_t,s_*. *x_t,s_* is the input of the grid cell (*t, s*), *h_t−1,s_* and *h_t,s−1_* are the output of grid cells (*t* − 1, *s*) and (*t*, *s* − 1). *I_t,s_* is the integrated matrix including the input of grid cell (*t, s*) *x_t,s_* and the output *h* of grid cell (*t* − 1, *s*) and (*t,s* − 1). *i_t,s_*, *f_t,s,1_*, *f_t,s,2_* and *o_t,s_* are the intermediate variables of grid cell (*t, s*).

$C_{t,s}$ can be transformed to the output $h_{t,s}$ that represents the traffic accident characteristics through an output rate $o_{t,s}$. $C_{t,s}$ can be interpreted as the traffic accidents potential. Within $C_{t,s}$, the elements corresponding to the accident counts, date, time, isolation and cross-sectional location can be viewed as the most dangerous location, date, time, isolation and cross-sectional location. If the second element in $C_{t,s}$ grows larger, the potential of the current grid cell will move to a date closer to winter, meaning the traffic accident will be more likely to happen in the winter.

The intermediate variables can be interpreted as follows. The $o_{t,s}$ shows the proportion of potential traffic accidents manifested as real traffic accidents. The $i_{t,s}$ shows the proportion of land use properties that affects the traffic accident characteristics, and the $f_{t,s,j}$ shows the proportion of surrounding traffic accident characteristics that generates an impact to the traffic accident characteristics of the current cell. In the training process of the model, although the intermediate variables are not directly determined, the basic parameters are weights and bias. Through these parameters, every grid cell resolves its own value of the intermediate variables $S_{t,s}$, $i_{t,s}$, $f_{t,s,j}$, and $o_{t,s}$:

*i_t,s_* = *σ*(*W_i_I_t,s_* + *b_i_*) (5)

*f_t,s,j_* = *σ*(*W_f,j_I_t,s_* + *b_f,j_*) (6)

*o_t,s_* = *σ*(*W_o_I_t,s_* + *b_o_*) (7)

where *W_i_* and *b_i_*, *W_f,j_* and *b_f,j_*, *W_o_* and *b_o_* are the weight matrix and bias matrix of the intermediated variables.

## 4. Discussion

The discussion section is organized as follow: Section 4.1 first presents the validation of the model comparing to the LSTM, RNN, and BPNN. This proves the effectiveness of the model and show its advantages over other neural network structures. Section 4.2 interprets the state $C_{t,s}$ of each grid cell to show the characteristics of traffic accident potential. The spatial aggregation of the traffic accident count, date, time, isolation and cross-sectional location are explained to discuss the accident potential. Section 4.3 detailed explains the intermediate variables in the urban safety context to reveal the influencing factors on these characteristics of traffic accident potential. The example conclusion can be drawn, as grid cells with higher $o_{t,s}$ are more likely for potential traffic accidents to occur. Section 4.4 summarizes the position of all grid cells, and some general rules are proposed based on weights and bias interpretations. The potential of accident date is found to be largely influenced by the local indicators; the potential of cross-sectional location is found to be less influenced by the local land use properties. 

Corresponding to the three levels, Section 4.2 focuses on the spatial distribution of the potential by explaining the accident potential. Section 4.3 focuses on an example grid cell by discussing the intermediate variables that influence the potential. Section 4.4 focuses on a general rule for the entire urban area through interpreting the weight matrix.

### 4.1. Validation of the MDLSTM Model

Before explaining the mechanism of the model, its accuracy and reliability are first tested in comparison with the other neural network models. In this section, backpropagation neural network (BPNN), recurrent neural network (RNN), long-short term memory neural network (LSTM), and the multi-dimensional long-short term memory neural network (MDLSTM) are used to show the differences in modeling the land use properties and accident characteristics. The results are as follows.

**Figure 9:**
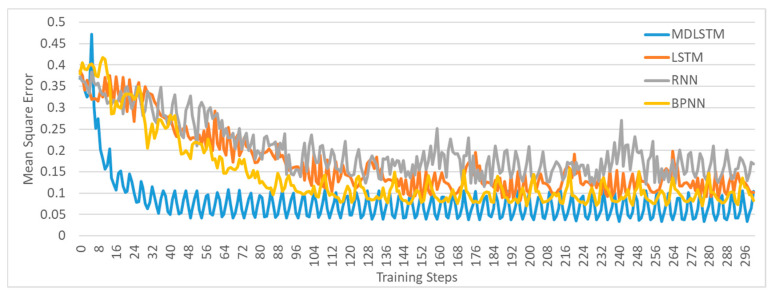
Performance of MDLSTM, LSTM, RNN and BPNN on the training dataset. MDLSTM means the “multi-dimensional long-short term memory neural network”. LSTM means the “long-short term memory neural network”. RNN means the “recurrent neural network”. BPNN means the “back-propagate neural network”.

Figure 9 shows the mean square error (MSE) of the MDLSTM, LSTM, RNN and BPNN models trained based on the training dataset. In the MDLSTM model, a 3 × 3 window, at the center of the 9 × 9 windows introduced in Section 3.1, is selected as the object for calculating MSE. This greatly reduces the impact of window sampling on the accuracy of the model. The windows are also applied in LSTM, RNN and BPNN models, so that the accuracy can be compared fairly. It shows that MDLSTM not only converges faster than the other three models on the training dataset, but also has a higher accuracy. In order to demonstrate whether the model is overfit, the performance of MDLSTM and LSTM, RNN, BPNN on the testing dataset are also compared. MDLSTM is proved to perform better, as shown in Table 4.

### 4.2. Characteristics of Traffic Accident Potential

It is known that the characteristics of traffic accident potential has a spatial distribution that disclose some significant, essential information about where or which kind of accidents could take place. 

According to the model structure, the potential $C_{t,s}$ of a grid cell is based on the input land use properties $x_{t,s}$, surrounding accident characteristics $h_{t-1,s}$ and $h_{t,s-1}$ and the intermediate variables $S_{t,s}$, $i_{t,s}$, $f_{t,s,j}$, and $o_{t,s}$. The accident characteristics $h_{t,s}$ can be determined by the potential $C_{t,s}$ and the intermediate variable $o_{t,s}$. 

**Figure 10:**
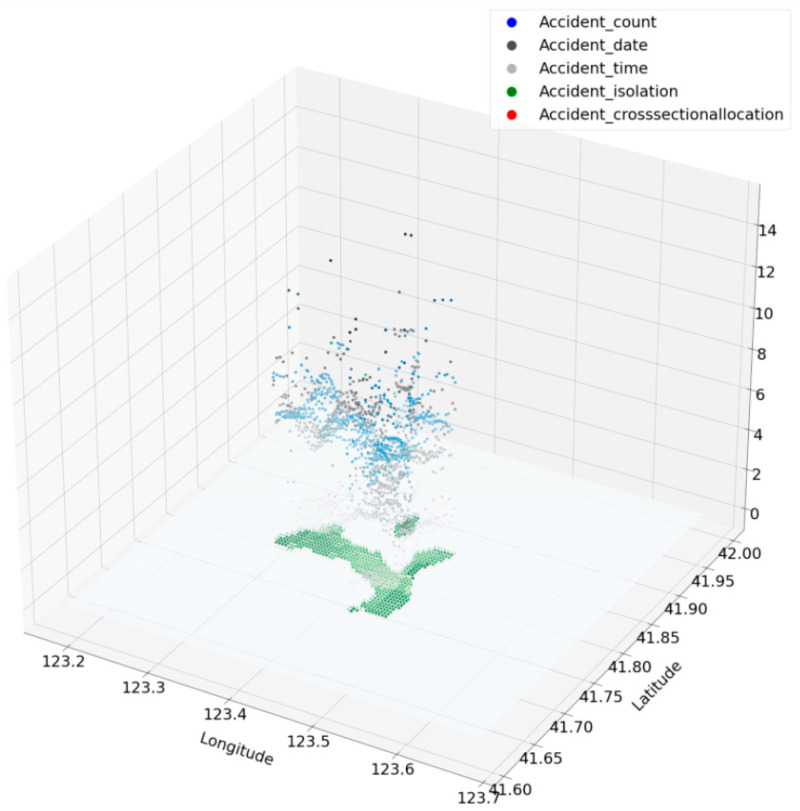
The spatial distribution of state value $C_{t,s}$.

In Figure 10, the two axes (length and width) indicates the spatial location of the grid cell, and the ordinate shows the size of the hidden danger of each traffic accident characteristics. For example, the higher black points are grid cells with high quality isolation, such as center isolation.

**Figure 11:**
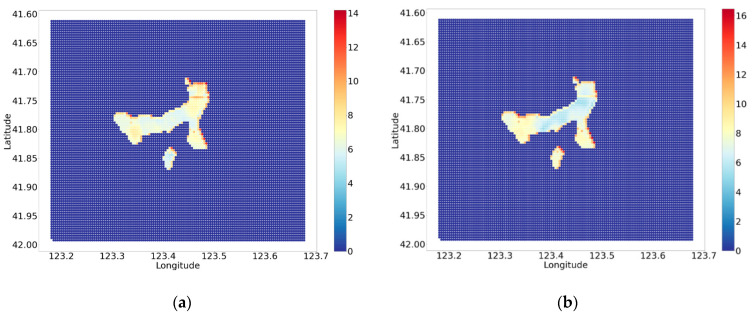
(**a**) The spatial distribution of potential of accident count. (**b**) The spatial distribution of date away from winter.

As for the accident count, the gathering area of traffic accident can be restricted to a certain area because of the training input and output data. However, comparing to the distribution of the accident, this area is much larger (see Figure 3). Considering the value of the accident count potential shown in Figure 11a, the accident count potential is gathered at several locations within the whole traffic accident potential area. Except for the scattered points, the horizontal line, which represents the 64th row, corresponds to the “Hunnan middle road”, where hidden dangers in traffic accident concentrate.

As for the accident date shown in Figure 11b, the dividing line of values lower than 10 and higher than 10 is at a similar position as the 64th row, which means that accidents are more likely to take place closer to winter on the north side of the line, and less likely on the south side of the line. In addition, the trend shows traffic accidents in the south-east of the urban center are more likely to occur in winter, and specific measures should be taken.

**Figure 12:**
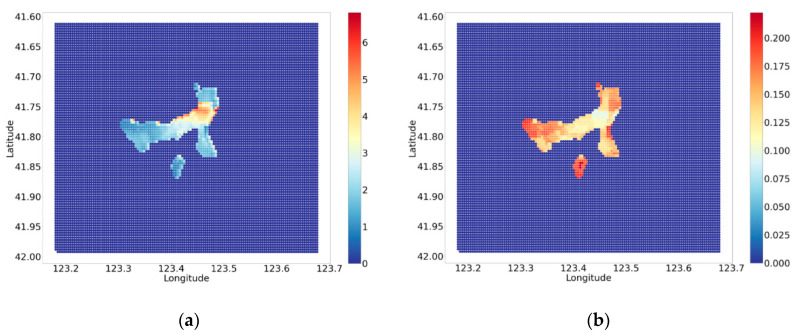
(**a**) The spatial distribution of the potential of accident time. (**b**) The spatial distribution of the isolation form.

As for the accident happening time, shown in Figure 12a, 15:00 is found to be the period of high traffic accidents (see Section 3.1.2), except in regions close to the dividing line. This indicates comparatively more accidents in the daytime. The north and west part of the urban area are also more dangerous at times. Since the isolation is decided by the presenting facilities, the results in Figure 12b only shows the distribution of the facilities, such as the isolation form of each road.

### 4.3. The Impact of Land Use Properties and Spatial Effect on the Traffic Accident

Summarizing the intermediate variables in the training dataset, the impact of the land use properties and the spatial effect on traffic accidents are analyzed. In this section, the values of intermediate variables of the grid cell (50, 40) are used as an example to show some local rules about the relationship between the land use properties and traffic accident characteristics. Then a surrounding region of the grid cell (50, 40) is discussed to show the regional similarities. This process and method could be promoted as a general method for urban safety and traffic accident investigation.

The value of intermediate variables of the grid cell (50, 40) (*t* = 3, *s* = 316) shown in the Table 5 brings several interesting insights. The element in the table shows the value of the intermediate variable corresponding to the accident characteristics.

For example, the first cell in this table, 0.3, means that 30% of the accident potential caused by the land use properties can join the potential calculation and be transferred to the final number of accidents. 0.12 in the first column and the second row shows every 1 unit change in land use properties will cause a 0.12 units change in land use potential, regardless of the $i_{t,s}$. The third and fourth value in the first column, 0.74 and 0.05, shows that 74% and 5% of the accident potential can be transferred from the north and west neighboring grid cells. 0.6 in the first column and the last row shows at least 60% of the potential of accident count will take place in reality. Since these variables are from either σ (1st, 3th, 4th and 5th row) function or tanh (2nd row) function, the 2nd row has both negative and positive values. 

For the grid cell (50, 40), among all accident characteristics, the accident date (0.77) has the highest proportion of accident potential depending on the land use properties. In contrast, the accident date is negatively affected by the land use properties (−0.27). The one with the highest conversion rate (0.97) are from land use properties to the potential of accident time. From the direction point of view, it is clear that the $f_{t,s,j}$ in the first dimension is much larger than in the second dimension. That may reflect the road form, since this point is near a high level vertical road. In addition, the $o_{t,s}$ shows that about 60% of the traffic accidents potential on the plot will cause accidents. The date and time of the accident are relatively closer (0.56 and 0.62, while 0 is the closest and 1 is the farthest) to winter and night.

**Figure 13:**
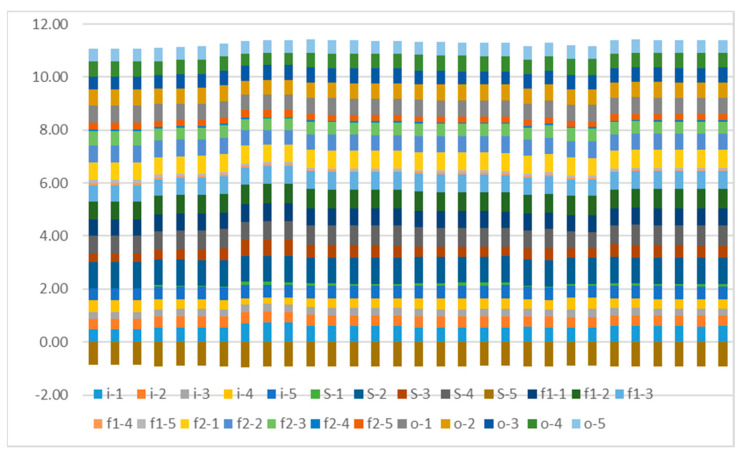
The values of intermediate variables coming from 30 continuous windows in a batch behind the grid cell (50, 40). Each color of the figure shows the corresponding value of the type of intermediate variable. For example, the “*i* − 1” variable shows the first element of the *i_t,s_*.

The regional regulation is based on the 30 usable windows behind the grid cell (50, 40), as shown in Figure 13. It shows the volatility and distribution of the intermediate variables in this region. In general, the intermediate variables have little change among the grid cells in this area.

### 4.4. General Rules Based on the Interpretation of the Weight Matrix

As discussed in Section 3.3, the weight matrix shows the basic rule that traffic accidents obey. By sorting out and summarizing the relationship of each grid cell among the land use properties, intermediate variables, traffic accident potential and traffic accident characteristics, a general rule can be devised.

#### 4.4.1. Relationship between Land Use Properties $x_{t,s}$ and Accident Potential $S_{t,s}$

$W_{C}$ plays an essential role in the model, since it transforms a grid cell’s land use properties to the accident potential. Meanwhile, $W_{C}$ itself is also generated based on the land use properties. In this section, elements in $W_{C}$ are first explored and interpreted to show the basic relationship between land use and traffic accident potential. Table 6 shows the $W_{C}$ after training.

Negative values in $W_{C}$ suggest land use properties contribute negatively to the traffic accident potential. For example, the most negative effect is the impact of umber of surrounding road sections on the accident cross-section location (−0.57), which indicates that higher accessibility may lead to a higher possibility for non-motorized lanes traffic accidents than in motorized lanes.

Positive values suggest the land use properties have a positive impact to the accident characteristics. For example, the most positive effect is congestion ratio on the accident date (2.11), which indicates that higher congested areas may lead to accident occurrences further away from winter.

The comparison of numbers in the “Sum” row indicates the relative impact of all the chosen land use properties on the accident potential. The accident date is found to be largely influenced by the land use properties (2.93), which shows great variations in the dates of accidents potential in different regions, and the need for more targeted measures in seasonal control. Every type of land use properties has a positive impact on the accident date, except for the distance to CBD. However, the accident cross-sectional location is negatively affected by the land use properties (−0.59).

#### 4.4.2. Accident Potential $C_{t,s}$ Based on the Local One $S_{t,s}$

The $i_{t,s}$ shows the proportion of local state $S_{t,s}$ that will influence the accident potential $C_{t,s}$. This $i_{t,s}$ is further generated based on the input weight $W_{i}$. Therefore, elements in $W_{i}$ reflects the impact of land use properties on the proportion of hidden traffic accidents caused by local land use properties. Table 7 shows $W_{i}$ after training.

In the $W_{i}$ which differs from $W_{C}$, negative values suggest the land use properties has lower contribution to the traffic accident potential. For example, the most negative effect is the impact of congestion ratio on the accident count (−1.34), which indicates the higher the level of congestion, the lower the number of accidents affected by local land use properties. Positive values, such as the congestion ratio on the accident date (3.24), indicates that the date of traffic accident occurrences in higher congested area may be further away from winter.

The comparison of numbers in the “Sum” row indicates the relative impact of all the chosen land use properties on the accident potential. In accordance to Equation (2), the impact of this $W_{i}$ is very similar to the $W_{C},$ since the accident potential is affected by the product of $i_{t,s}$ and $S_{t,s}$.

#### 4.4.3. Accident Potential Transfer from the Neighboring Grid Cells $C_{t-1,s}$ and $C_{t,s-1}$

As discussed in Section 3.3., $W_{f,j}$ represents the impact of land use properties on the transferred accident potential from the neighboring grid cells. This is the main step for considering the spatial effects in the model. Since we suppose the unit values of the spatial effect in east-west direction and the north-south direction are equivalent, the focus is placed on $W_{f,1\&2}$, the sum of $W_{f,1}$ and $W_{f,2}$. Table 8 shows the first part of the transfer weight matrix $W_{f,1\&2}$ after training.

In the $W_{f,1\&2}$, negative values suggest the land use properties has lower contribution to the transfer ratio of traffic accident potential. For example, the most negative effect is the impact of congestion ratio on the accident count (−1.55), which indicates the higher the level of congestion, the lower the number of accidents affected by surrounding accident potential. Positive values, such as the congestion ratio on the accident isolation (4.15), indicates that the isolation form is affected more by the neighboring grid cells.

Moreover, the comparison between numbers in the “Sum” row shows a similar conclusion that the potential of accident count is less influenced by the neighboring accident potential. The 3.04, corresponding to the accident isolation, shows that the isolation form of every grid cell is greatly influenced by the spatial effect.

#### 4.4.4. Proportion of Accident Potential $C_{t,s}$ That Leads to an Accident $h_{t,s}$

The $o_{t,s}$ represents the proportion of accident potential, $C_{t,s}$, that leads to an accident, $h_{t,s}$. $W_{o}$, as the corresponding weight, represents the impact of land use properties on $o_{t,s}$. Higher $o_{t,s}$ suggests a serious proportion of accidents potential resulting in traffic accidents, which also reflect factors in the local area for avoiding traffic accidents. Table 9 shows the first part of the input weight matrix $W_{o}$ after training.

In $W_{o}$, negative values also suggest the land use properties has lower contribution to the $o_{t,s}$, which shows the proportion of traffic accident potential that eventually occurred. For example, the most negative effect is the impact of distance to CBD on accident isolation (−1.07), which indicates that the farther away from CBD, the less likely for the isolation of hidden traffic accident to result in accidents. Positive values, such as the distance to CBD on the accident cross-section location (0.64), indicates that the farther away from CBD, the more significant the cross-sectional location becomes as a factor in the potential of traffic accident that result in accidents. 

Moreover, the comparison between numbers in the “Sum” row shows that the accident time will be far from potential if the land use properties gets higher. The accident count will also be more predictable and explainable by the traffic accident potential with a higher land use property.

## 5. Conclusions

This study focuses on the interpretation and application of the multi-dimensional long-short term memory neural network model (MDLSTM) on modelling the relationship between traffic accident and selected land use properties. 

The idea is to divide the influencing factors of traffic accident into two categories: a spatial category and a local category. The local category considers land use properties, which include the plot ratio, number of types of POIs, centrality, distance to CBD, number of surrounding road sections and congestion ratio. Other parameters are considered in the spatial category.

Some interesting insights are found. (1) The spatial distribution of accident potential purposed a division line, on both side of which the accident potential shares significant differences. (2) Spatial effect differ strongly through directions between north-to-south and west-to-east, especially the characteristics about the physical infrastructure, such as the isolation form. (3) The potential of accident date is found to be largely influenced by the local indicators, and the potential of cross-sectional location is found to be less affected by the local land use properties. The potential of isolation form is highly spatial correlated, while the accident count shows differences. As for the proportion of potential accident that causes real-life traffic accident, the accident count shows better interpretability, while the higher land use characteristics leads to lower accuracy in accident time prediction.

Based on the findings above, several applicable advices can be proposed to the urban managers and researchers. It is a practical problem for urban managers to predict the location of the traffic accidents, especially for managers in Shenyang. Results show that “Hunnan middle road” is an essential accident potential hotspot. It also illustrates the potential form which further shows the accidents might be a critical problem in some regions near city center. At the level of the whole urban area, focus need to be put on the accident non-motorized lane especially in the suburban area with simple isolation facilities. In addition, the traffic accidents around congested area are also important since it positively correlated to the plot ratio. Winter accidents may occur far away from the city center. Therefore, target measures are needed in seasonal accident control.

The innovations of this paper are: 1. Multiple local and surrounding influence factors are considered, and appropriate model is used to capture their influence. The model separates spatial influence factors from local influence factors, which greatly improves the interpretability of traffic accident analysis models. 2. Multi-Dimensional Long Short Term neural network (MDLSTM) model is used to explore the relationship between input and output, with higher accuracy and computational efficiency. 3. Interpretation of the relationship of land use properties and traffic accidents are proposed, and a three levels of explanation method was used. The hidden factor-accident potential is found, containing the local and spatial effect. At last, the general rules of land use properties with the traffic accident characteristics are interpreted in detail to provide guidance for policy making.

## Figures and Tables

**Table 1:** The overview of the land use properties data.

Land Use Properties	Unit	Data Range			
		Minimum (Min)	Maximum (Max)	Mean	Standard Error (Std)
Plot ratio	-	0	6.62	0.341	0.777
Number of types of POIs *	-	2.00	13.0	4.39	1.92
Centrality	m (meter)	4.49 × 10^3^	3.37 × 10^4^	1.31 × 10^4^	5.60 × 10^3^
Distance to CBD *	m	643	3.21 × 10^4^	1.00 × 10^3^	5.53 × 10^3^
Number of surrounding road sections	-	0	221	34.1	34.1
Congestion ratio	%	0	0.486	0.00245	0.0227

* POI means “point of interest” and CBD means “central business district”.

**Table 2:** The overview of the accident characteristics.

Traffic Accident Characteristics	Unit	Data Range			
		Min	Max	Mean	Std
Accident count	-	0	45	11.4	9.39
Accident date	d (day)	0	183	117	52.2
Accident time	s (second)	300	8.62 × 104	4.54 × 104	2.33
Accident isolation	-	0	3	0.490	0.893
Accident cross-sectional location	-	0	5	4.60	0.957

**Table 3:** Global Moran’s I and Global Geary’s C spatial dependency test.

**Global Moran’s I**		**0.128**
I	*p*-value	1.76 × 10 − 10
	z-score	6.38
**Global Geary’s C**		**0.868**
C	*p*-value	0.000171
	z-score	−3.58

**Table 4:** Performance of MDLSTM, LSTM, RNN and BPNN on the testing dataset.

Testing Indicator	MDLSTM	LSTM	RNN	BPNN
Mean squared error of the whole test dataset	0.16	0.27	0.30	0.34

**Table 5:** The value of intermediate variables of the grid cell (50, 40).

Intermediate Variable	Accident Count	Accident Date	Accident Time	Accident Isolation	Accident Cross-Section Location
$i_{t,s}$	0.30	0.77	0.41	0.29	0.23
$S_{t,s}$	0.12	−0.27	0.97	0.61	0.70
$f_{t,s,1}$	0.74	0.65	0.26	0.59	0.66
$f_{t,s,2}$	0.05	0.05	0.07	0.08	0.06
$o_{t,s}$	0.60	0.56	0.62	0.45	0.47

**Table 6:** $W_{C}$ after training.

Key Features	Accident Count	Accident Date	Accident Time	Accident Isolation	Accident Cross-Section Location
Plot ratio	−0.39	0.13	0.09	0.00	−0.49
Number of types of POIs	−0.12	0.32	0.37	0.25	−0.27
Centrality	−0.12	0.22	0.32	−0.28	0.04
Distance to CBD	0.41	−0.24	0.30	0.19	0.49
Number of surrounding road sections	−0.49	0.39	0.08	0.04	−0.57
Congestion ratio	1.02	2.11	−0.73	1.66	0.21
Sum	0.31	2.93	0.42	1.86	−0.59

**Table 7:** $W_{i}$ after training.

Key Features	Accident Count	Accident Date	Accident Time	Accident Isolation	Accident Cross-Section Location
Plot ratio	0.19	0.09	−0.03	0.31	0.27
Number of types of POIs	0.00	−0.27	0.01	0.07	−0.99
Centrality	0.03	0.34	0.16	−0.26	0.64
Distance to CBD	0.71	−0.85	1.09	−0.50	1.61
Number of surrounding road sections	−0.01	0.13	−0.03	0.13	−0.23
Congestion ratio	−1.34	3.24	0.62	1.15	−2.05
Sum	−0.43	2.69	1.82	0.89	−0.74

**Table 8:** $W_{f,1\&2}$ after training.

Key Features	Accident Count	Accident Date	Accident Time	Accident Isolation	Accident Cross-Section Location
Plot ratio	−0.43	−0.26	−0.35	0.90	−0.05
Number of types of POIs	0.04	0.02	0.39	0.99	−0.15
Centrality	−0.87	0.72	−0.18	−1.35	−0.48
Distance to CBD	0.22	0.13	0.01	−0.95	−0.36
Number of surrounding road sections	0.01	0.60	0.15	−0.71	0.17
Congestion ratio	−1.55	−0.14	−0.86	4.15	−0.10
Sum	−2.58	1.08	−0.84	3.04	−0.97

**Table 9:** $W_{o}$ after training.

Key Features	Accident Count	Accident Date	Accident Time	Accident Isolation	Accident Cross-Section Location
Plot ratio	0.08	0.23	0.21	−0.05	−0.29
Number of types of POIs	−0.13	−0.01	0.16	−0.15	0.13
Centrality	−0.17	0.01	−0.42	0.44	−0.35
Distance to CBD	0.20	0.09	−0.70	−1.07	0.64
Number of surrounding road sections	0.39	0.03	−0.38	0.15	−0.47
Congestion ratio	0.27	−0.29	−0.42	−0.26	−0.16
Sum	0.64	0.06	−1.55	−0.94	−0.50

## Data Availability

Not applicable.
